# Emergency department use by persons with MS: A population-based descriptive study with a focus on infection-related visits

**DOI:** 10.1177/13524585221078497

**Published:** 2022-03-01

**Authors:** Jonas Graf, Huah Shin Ng, Feng Zhu, Yinshan Zhao, José MA Wijnands, Charity Evans, John D Fisk, Ruth Ann Marrie, Helen Tremlett

**Affiliations:** Division of Neurology, Department of Medicine, Faculty of Medicine, Djavad Mowafaghian Centre for Brain Health, UBC Hospital, The University of British Columbia, Vancouver, BC, Canada; Division of Neurology, Department of Medicine, Faculty of Medicine, Djavad Mowafaghian Centre for Brain Health, UBC Hospital, The University of British Columbia, Vancouver, BC, Canada; Division of Neurology, Department of Medicine, Faculty of Medicine, Djavad Mowafaghian Centre for Brain Health, UBC Hospital, The University of British Columbia, Vancouver, BC, Canada; Division of Neurology, Department of Medicine, Faculty of Medicine, Djavad Mowafaghian Centre for Brain Health, UBC Hospital, The University of British Columbia, Vancouver, BC, Canada; Division of Neurology, Department of Medicine, Faculty of Medicine, Djavad Mowafaghian Centre for Brain Health, UBC Hospital, The University of British Columbia, Vancouver, BC, Canada; College of Pharmacy and Nutrition, University of Saskatchewan, Saskatoon, SK, Canada; Nova Scotia Health Authority, Departments of Psychiatry, Psychology and Neuroscience, and Medicine, Dalhousie University, Halifax, NS, Canada; Departments of Internal Medicine and Community Health Sciences, Max Rady College of Medicine, Rady Faculty of Health Sciences, University of Manitoba, Winnipeg, MB, Canada; Division of Neurology, Department of Medicine, Faculty of Medicine, Djavad Mowafaghian Centre for Brain Health, UBC Hospital, The University of British Columbia, Vancouver, BC, Canada

**Keywords:** Multiple sclerosis, emergency department, healthcare utilization, population-based data

## Abstract

We described emergency department (ED) visits (all visits and infection-related) by persons with multiple sclerosis (MS) in British Columbia, Canada (1 April 2012 to 31 December 2017). We identified 15,350 MS cases using health administrative data; 73.4% were women, averaging 51.4 years at study entry. Over 4.9 years of follow-up (mean), 56.0% of MS cases visited an ED (mean = 0.6 visits/person/year; total = 37,072 visits). A diagnosis was documented for 25,698 (69.3%) ED visits, and 18.4% (4725/25,698) were infection-related. Inpatient admissions were reported for 20.4% (5238/25,698) of all and 29.2% (1380/4725) of infection-related ED visits. Findings suggest that the ED plays a substantial role in MS healthcare and infection management.

## Introduction

Emergency department (ED) presentations represent an important aspect of health care utilization in the general population.^1,2^ Compared to the general population, persons with multiple sclerosis (MS) frequently access the healthcare system, with infections contributing to this higher healthcare use. For example, persons with MS had 41% more infection-related physician claims (adjusted rate ratio = 1.41; 95% confidence interval: 1.36–1.47) versus a sex-, age-, and region-matched non-MS population.^3^ However, relatively little is known about ED utilization by persons with MS.^4–6^ Here, we described overall and infection-related ED use in an MS population.

## Methods

We performed a descriptive, population-based study in British Columbia (BC), Canada, using linked health administrative data (via Population Data BC^7^), including Medical Service Plan Billing Information^8^ (providing physician claims); the Discharge Abstract Database^9^ (providing hospital admissions/discharges); PharmaNet^10^ (for prescriptions filled at outpatient/community pharmacies); Census Geodata (providing socioeconomic status estimates); Registration and Premium Billing files^11^ (providing BC residency status via the mandatory healthcare plan registration days); Vital Statistics^12^ (capturing death dates); and the National Ambulatory Care Reporting System dataset^13^ (providing ED-related visit dates and Diagnosis Shortlist codes,^14^ including infections; Supplementary Table 1) starting 1 April 2012).

All MS cases were identified with a validated algorithm.^15^ Study entry date was the later of the first MS/demyelinating disease-related International Classification of Diseases (ICD-9/10) code, or first disease-modifying drug (DMD) prescription filled, or 1 April 2012 (start of the ED date). All included persons were ⩾18 years old and BC residents for ⩾1 year pre-study entry; follow-up ended at the earliest of death, emigration, or 31 December 2017. Comorbidities were measured using a modified Charlson Comorbidity Index during the 1-year pre-study entry.^16–18^ MS cases ever filling a DMD prescription during follow-up were described.

## Results

We identified 15,350 MS cases (11,270 (73.4%) women; Table 1), of whom 86.5% (13,274/15,350) entered the study in 2012/2013, as per data availability. At study entry, the mean age was 51.4 years (standard deviation (SD) =13.8), and 26.5% (4072/15,350) had ⩾1 comorbidity. During the mean 4.9 (SD = 1.6) years of follow-up, 56.0% (8603/15,350) of MS cases visited an ED, totaling 37,072 visits; 15,350 MS cases averaged 0.6 (SD = 1.6) visits/person/year. MS cases visiting an ED at least once during follow-up (*n* = 8603) with (vs without) comorbidity at study entry were older (mean age = 56.5 (SD = 14.3) vs 49.5 (SD = 13.7) years) and averaged more ED visits/person/year (1.3 (SD = 2.4) vs 0.9 (SD = 1.9)). More than a quarter of MS cases had ⩾3 ED visits (4064/15,350; 26.5); of these 1383 (34.0%) of 4064 had ⩾1 comorbidity at study entry. Most ED visits by MS cases did not require an ambulance (26,359/37,072; 71.1%); 28.8% required a ground ambulance (10,691/37,072) and >95% were of semi-urgent or higher priority (35,468/37,072).

**Table 1.** Characteristics of the multiple sclerosis study population in British Columbia, Canada (2012–2017).

Characteristics at the index date	Total, *n* = 15,350
Sex, *n* (%)	
Women	11,270 (73.4)
Men	4080 (26.6)
Age at study entry in years	
Mean (SD)	51.4 (13.8)
Age group at study entry, *n* (%)	
<30 years	1017 (6.6)
30–39 years	2137 (13.9)
40–49 years	3495 (22.8)
50–59 years	4339 (28.3)
⩾60 years	4362 (28.4)
Calendar year at study entry,^a^ *n* (%)	
2012–2013	13,274 (86.5)
2014–2015	1203 (7.8)
2016–2017	873 (5.7)
Socioeconomic status,^b^ *n* (%)	
1 (lowest income quintile)	2903 (18.9)
2	2905 (18.9)
3	3048 (19.9)
4	3302 (21.5)
5 (highest income quintile)	3043 (19.8)
Unavailable	149 (1.0)
Comorbidity score,^c^ *n* (%)	
0	11,278 (73.5)
1	2497 (16.3)
2	990 (6.4)
⩾3	585 (3.8)
Characteristics over the study follow-up	
Follow-up^a^ time in years	
Mean (SD)	4.9 (1.6)
Filled ⩾1 DMD prescription at any time during follow-up,^d^ *n* (%)	3079 (20.3)
• *First-generation DMD—any*	*2190/3079 (71.1)*
• *Second generation DMD—any*	*1716/3079 (55.7)*
Visits to the ED, no. of persons with MS (%)	
Never	6747 (43.9)
Once only	2849 (18.6)
Twice only	1690 (11.0)
⩾3 times^e^	4064 (26.5)
Total number of ED visits	37,072
Mode of transportation, *n* (%)^e^	
No ambulance	26,359 (71.1)
Ground ambulance only	10,691 (28.8)
Air ambulance only	15 (<0.1)
Combination of air and ground ambulance	7 (<0.1)
Characteristics over the study follow-up	
Triage level received, *n* (%)^e^	
Resuscitation	294 (0.8)
Emergent	6145 (16.6)
Urgent	18,689 (50.4)
Semi-urgent	10,340 (27.9)
Non-urgent	1378 (3.7)
Unknown	226 (0.6)

DMD: disease-modifying drugs; N/A: not applicable; SD: standard deviation; ED: emergency department.

a Follow-up was from study entry to end. As per data availability, the earliest possible study entry was 1 April 2012.

b Socioeconomic status is represented by neighborhood income quintiles, based on the closest available measurement to the study entry date.

c Comorbidity was measured using the Charlson Comorbidity Index (modified to exclude hemiplegia/paraplegia to avoid misclassifying MS complications) during the 1-year period prior to the study entry date.^17,18^ The proportion of persons with MS scoring 1 or more on the Comorbidity Index is consistent with prior work^16^ conducted in similar cohorts. The most common comorbid conditions (present at study entry) which were identified using the Index were “chronic pulmonary disease” (present in 1102/15,350; 7.2% of the study cohort), “diabetes mellitus without chronic complications” (1011; 6.6%), and “cerebrovascular disease” (629; 4.1%).

d Captured as prescriptions filled; some people were exposed to >1 DMD during follow-up; hence, the sum of cases filling first- and second-generation DMD prescriptions exceeded the sum of cases ever filling a DMD prescription; first-generation DMDs included beta-interferon and glatiramer acetate, and second-generation DMDs included natalizumab, fingolimod, dimethyl fumarate, teriflunomide, alemtuzumab, daclizumab, and ocrelizumab. Pre-study entry (1 January 1996 to 31 March 2012) 19.5% (2991/15,350) of MS cases had ever filled a DMD prescription.

e The denominator used to estimate the following proportions was the total number of ED visits captured during follow-up (*n* = 37,072); some people had more than one ED visit during follow-up; hence, the sums of ED visits exceed the total number of persons with at least one ED visit.

### ED visits with a known diagnosis

Diagnostic codes were reported for 69.3% (25,698/37,072) of ED visits (summarized in Supplementary Table 2). These visits were made by 74.2% (6384/8603) of MS ED users. Of these, the most frequent primary diagnoses were “abdominal pain/colic” (1279/25,698 visits; 5.0%), “urinary tract infection” (1277 visits; 5.0%), and “MS” (1225 visits; 4.8%). When combined, 18.4% (4725/25,698) of ED visits were infection-related with 32.2% (2056/6384) of MS participants having at least one such visit. Nearly one-third (1380/4725; 29.2%) of infection-related ED visits led to hospitalization, while 20.4% (5238/25,698) of all ED visits made by 27.9% (2404/8603) of cases did so (Supplementary Tables 2 and 3).

### DMD users versus non-users

Persons who filled ⩾1 DMD prescription(s) during follow-up (“DMD users”) were younger at study entry date than non-users (mean age = 41.9 (SD = 11.1) vs 53.8 (SD = 13.3) years). However, the proportions accessing an ED at least once were generally similar (1806/3079, 58.0% of DMD users and 6797/12,271, 55.6% of non-user), as was the study follow-up time (4.8 years (SD = 1.6) for DMD users vs 4.9 (SD = 1.6) for non-users).

### Socioeconomic status

There were no clear patterns across the socioeconomic quintiles at study entry for the MS cases: ever/never filling a DMD prescription during follow-up, ever/never being hospitalized subsequent to an ED visit, or for the five most common ED diagnoses (data not shown).

## Conclusion

Over 50% of persons with MS had more than one ED consultation during our nearly 6-year observation period; one-quarter visited an ED three or more times. Nearly 20% of ED visits were infection-related; one-third resulted in hospitalization, whereas one-fifth of all-cause ED visits did so. MS cases with ⩾1 (vs without) comorbidity at study entry averaged more ED visits/person/year. Our intentionally descriptive study has several limitations. While the overall burden of infection-related ED visits was considerable in our MS population, this could be higher as diagnoses were unavailable for one-third of all ED visits. Furthermore, our study lacked MS-specific clinical information, such as relapses, and a comparison to the general population. Our 1-year pre-study lookback period may have reduced the detection of comorbidities. Strengths of our study included the large cohort of MS cases identified using a validated algorithm within a geographically defined population with universal healthcare coverage, including ED visits. Our results suggest that the ED visits by MS cases often lead to hospitalizations, perhaps more so for infection-related visits. Further studies are necessary to better understand the importance of ED use by persons with MS.

## Supplemental Material

sj-docx-1-msj-10.1177_13524585221078497 – Supplemental material for Emergency department use by persons with MS: A population-based descriptive study with a focus on infection-related visits Click here for additional data file. Supplemental material, sj-docx-1-msj-10.1177_13524585221078497 for Emergency department use by persons with MS: A population-based descriptive study with a focus on infection-related visits by Jonas Graf, Huah Shin Ng, Feng Zhu, Yinshan Zhao, José MA Wijnands, Charity Evans, John D Fisk, Ruth Ann Marrie and Helen Tremlett in Multiple Sclerosis Journal

sj-docx-2-msj-10.1177_13524585221078497 – Supplemental material for Emergency department use by persons with MS: A population-based descriptive study with a focus on infection-related visits Click here for additional data file. Supplemental material, sj-docx-2-msj-10.1177_13524585221078497 for Emergency department use by persons with MS: A population-based descriptive study with a focus on infection-related visits by Jonas Graf, Huah Shin Ng, Feng Zhu, Yinshan Zhao, José MA Wijnands, Charity Evans, John D Fisk, Ruth Ann Marrie and Helen Tremlett in Multiple Sclerosis Journal

sj-docx-3-msj-10.1177_13524585221078497 – Supplemental material for Emergency department use by persons with MS: A population-based descriptive study with a focus on infection-related visits Click here for additional data file. Supplemental material, sj-docx-3-msj-10.1177_13524585221078497 for Emergency department use by persons with MS: A population-based descriptive study with a focus on infection-related visits by Jonas Graf, Huah Shin Ng, Feng Zhu, Yinshan Zhao, José MA Wijnands, Charity Evans, John D Fisk, Ruth Ann Marrie and Helen Tremlett in Multiple Sclerosis Journal
