# Supplementary material for: Emergency department use by persons with MS: A population-based descriptive study with a focus on infection-related visits
Source: Mult Scler. 2022 Mar 1;28(11):1825–8. doi: 10.1177/13524585221078497 (PMC9442277; doi:10.1177/13524585221078497)
Supplement: sj-docx-1-msj-10.1177_13524585221078497 – Supplemental material for Emergency department use by persons with MS: A population-based descriptive study with a focus on infection-related visits [file sj-docx-1-msj-10.1177_13524585221078497.docx]

**Supplementary Table 1:** The Canadian Emergency Department Diagnosis Shortlist codes indicative for an infection-related emergency department encounter.

| **Code** | **Disease** |
| --- | --- |
| A047 | Clostridium difficile |
| A051 | Botulism |
| A059 | Bacterial foodborne intox |
| A099 | GE - Gastroenteritis / Diarrhea |
| A1691 | TB - Tuberculosis |
| A35 | Tetanus |
| A379 | Pertussis / Whooping cough |
| A38 | Scarlet fever |
| A390 | Meningitis, meningococcal |
| A392 | Meningococcemia, acute |
| A419 | Septicemia |
| A46 | Erysipelas |
| A480 | Gas gangrene |
| A481 | Legionnaire's disease |
| A483 | TSS - Toxic shock syndrome |
| A499 | Bacteremia |
| A549 | Gonorrhea |
| A630 | Genital warts |
| A64 | Sexually transmitted infection |
| A692 | Lyme disease |
| A86 | Encephalitis, viral |
| A879 | Meningitis, viral |
| B009 | Herpes |
| B019 | Chickenpox / Varicella |
| B029 | Shingles / Zoster |
| B059 | Measles |
| B069 | Rubella / German measles |
| B083 | 5th disease |
| B084 | Hand, foot and mouth disease |
| B09 | Exanthema, viral |
| B199 | Hepatitis, viral |
| B24 | HIV |
| B269 | Mumps / Parotitis |
| B279 | Mononucleosis, infectious |
| B349 | Viral infection |
| B379 | Candidiasis |
| B49 | Tinea / Fungal infection |
| B54 | Malaria |
| B589 | Toxoplasmosis |
| B839 | Pinworms / Helminthiasis |
| B852 | Lice / Pediculosis |
| B86 | Scabies |
| B89 | Parasitic disease |
| G009 | Meningitis, bacterial |
| G039 | Meningitis, other |
| G060 | Intracranial abscess |
| G061 | Intraspinal abscess |
| G062 | Extradural / Subdural abscess |
| H029 | Stye / Chalazion / Blepharitis |
| H609 | OE - Otitis externa |
| H669 | OM - Otitis media |
| H709 | Mastoiditis |
| J019 | Sinusitis, acute |
| J029 | Pharyngitis, acute |
| J039 | Tonsillitis, acute |
| J040 | Laryngitis, acute |
| J041 | Tracheitis, acute |
| J042 | Laryngotracheitis, acute |
| J050 | Croup - A obstruct laryngitis |
| J051 | Epiglottitis, acute |
| J069 | URTI |
| J118 | Influenza / Flu syndrome |
| J189 | Pneumonia |
| J209 | Bronchitis, acute |
| J219 | Bronchiolitis, acute |
| J329 | Sinusitis, chronic |
| J36 | Peritonsillar abscess |
| J390 | Retro / Parapharyngeal abscess |
| J440 | COPD with respiratory infection |
| K047 | Dental / Periapical abscess |
| K612 | Anorectal abscess |
| K613 | Ischiorectal abscess |
| L010 | Impetigo |
| L029 | Abscess / Furuncle / Carbuncle |
| L0300 | Paronychia finger |
| L0301 | Paronychia toe |
| L039 | Cellulitis |
| L050 | Pilonidal cyst with abscess |
| M0099 | Arthritis, septic |
| M8699 | Osteomyelitis |
| N10 | Pyelonephritis |
| N12 | Tubulo-interstitial nephritis |
| N341 | Nonspecific urethritis |
| N390 | UTI - Urinary tract infection |
| N410 | Prostatitis, acute |
| N4592 | Orchitis / Epididymitis |
| N751 | Bartholin's abscess |
| N760 | Vaginitis, acute |
