# Supplementary material for: Emergency department use by persons with MS: A population-based descriptive study with a focus on infection-related visits
Source: Mult Scler. 2022 Mar 1;28(11):1825–8. doi: 10.1177/13524585221078497 (PMC9442277; doi:10.1177/13524585221078497)
Supplement: sj-docx-2-msj-10.1177_13524585221078497 – Supplemental material for Emergency department use by persons with MS: A population-based descriptive study with a focus on infection-related visits [file sj-docx-2-msj-10.1177_13524585221078497.docx]

**Supplementary Table 2:** Overview of the most common^a^ primary diagnostic codes^b^ of the n=25,698 emergency department (ED) visits in the multiple sclerosis population with a known ED-related diagnosis and the n=5,238 ED visits with a known ED-related diagnosis leading to hospitalization.

| **Code^b^** | **Description** | **Visits to the ED with known diagnosis, n (%^c^)** | **Visits to the ED with a known diagnosis leading to hospitalization, n (%^d^)** |
| --- | --- | --- | --- |
| R104 | Abdominal pain/colic | 1,279 (5.0) | 136 (2.6) |
| N390 | Urinary tract infection | 1,277 (5.0) | 335 (6.4) |
| G35 | MS | 1,225 (4.8) | 308 (5.9) |
| R074 | Chest pain | 1,083 (4.2) | 77 (1.5) |
| L039 | Cellulitis | 946 (3.7) | 86 (1.6) |
| Z5188 | Medical care, other | 858 (3.3) | 61 (1.2) |
| M545 | Back pain | 655 (2.5) | 42 (0.8) |
| J189 | Pneumonia | 473 (1.8) | 325 (6.2) |
| A419 | Septicemia | 450 (1.8) | 434 (8.3) |
| R53 | Weakness / Fatigue | 423 (1.6) | 187 (3.6) |
| R51 | Headache | 360 (1.4) | 20 (0.4) |
| A099 | GE - Gastroenteritis / Diarrhea | 311 (1.2) | 48 (0.9) |
| R69 | Diagnosis N/A in list | 306 (1.2) | 49 (0.9) |
| R55 | Syncope / Vasovagal | 257 (1.0) | 48 (0.9) |
| Z459 | Adjustment implanted device | 250 (1.0) | 6 (0.1) |

Key:

^a^‘Common’ was defined as codes which were reported in at least n=250 ED visits of the n=25,698 ED visits with a known diagnosis (≥1%).

Other ED-related codes of interest that represented <1% of all such codes reported for ED visits with a known diagnosis (n=25,698) included: diagnoses ‘social problem’ (code: Z659), ‘encephalopathy’ (code: G395), ‘hypertensive encephalopathy’ (code: I674) and ‘cachexia / failure to thrive’ (code: R64) were not common. For example, only a small proportion of ED visits were due to a ‘social problem’ (0.1%) or ‘cachexia / failure to thrive’ (0.1%).

^b^The Canadian Emergency Department Diagnoses Shortlist (CED-DxS), https://secure.cihi.ca/estore/productSeries.htm?locale=en&pc=PCC515&_ga=2.5501683.1173736235.1632862164-705063216.1632862164 (2021, accessed 28 September 2021).

^c^Denominator for the percentages shown = 25,698; representing all ED visits with a known diagnosis

^d^Denominator for the percentages shown = 5,238; representing all ED visits with a known diagnosis that led to a hospitalization
