# Supplementary material for: Emergency department use by persons with MS: A population-based descriptive study with a focus on infection-related visits
Source: Mult Scler. 2022 Mar 1;28(11):1825–8. doi: 10.1177/13524585221078497 (PMC9442277; doi:10.1177/13524585221078497)
Supplement: sj-docx-3-msj-10.1177_13524585221078497 – Supplemental material for Emergency department use by persons with MS: A population-based descriptive study with a focus on infection-related visits [file sj-docx-3-msj-10.1177_13524585221078497.docx]

**Supplementary Table 3:** Characteristics of the hospitalized multiple sclerosis cases subsequent to an emergency department visit with a known diagnosis; study population in British Columbia, Canada (2012-2017).

| **Characteristics at study entry** | **MS cases hospitalized due to ED visit with a known diagnosis, n=2,404** |
| --- | --- |
| **Sex, n (%)**  Women  Men | 1,635 (68.0)  769 (32.0) |
| **Age at study entry in years,**  mean (SD) | 55.6 (14.8) |
| **Age group at study entry, n (%)**  < 30 years  30 to 39 years  40 to 49 years  50 to 59 years  ≥60 years | 135 (5.6)  216 (8.9)  440 (18.3)  623 (25.9)  990 (41.2) |
| **Calendar year at study entry^a^, n (%)**  2012-2013  2014-2015  2016-2017 | 2,141 (89.1)  166 (6.9)  97 (4.0) |
| **Socioeconomic status^b^, n (%)**  1 (lowest income quintile)  2  3  4  5 (highest income quintile)  Unavailable | 536 (22.3)  467 (19.4)  433 (18.0)  491 (20.4)  447 (18.6)  30 (1.3) |
| **Comorbidity score^c^, n (%)**  0  1  2  ≥3 | 1,458 (60.6)  505 (21.0)  247 (10.3)  194 (8.1) |
| **Characteristics over the study follow-up** |  |
| **Follow-up^a^ time in years,**  mean (SD) | 4.9 (1.5) |
| **Filled ≥1 DMD prescription at any time during follow-up^d^, n (%)**  *- first generation DMD – any*  *- second generation DMD – any* | 379 (15.8)  *258/379 (68.1)*  *228/379 (60.2)* |
| **Visits to the ED, n of persons with MS (%)**  Never  Once only  Twice only  ≥3 times | 0  347 (14.4)  348 (14.5)  1,709 (71.1) |
| **Number of ED visits leading to hospitalization**  **Mode of transportation, n (%)^e^**  No ambulance  Ground ambulance only  Air ambulance only  Combination of air and ground ambulance  **Triage level received, n (%)^e^**  Resuscitation  Emergent  Urgent  Semi-urgent  Non-urgent  Unknown  **ED visit with diagnosis ‘MS’, n (%)**  **ED visit with diagnosis other than ‘MS’, n (%)** | **5,238**  2,093 (40.0)  3,139 (59.9)  <6  <6  172 (3.3)  1,848 (35.3)  2,831 (54.0)  337 (6.4)  24 (0.5)  26 (0.5)  308 (5.9)  4,930 (94.1) |

Key: DMD, disease-modifying drugs; N/A, not applicable; SD, standard deviation; ED, emergency department.

^a^Follow-up was from study entry to end. As per data availability, the earliest possible study entry was 1-April-2012.

^b^Socioeconomic status is represented by neighbourhood income quintiles, based on the closest available measurement to the study entry date.

^c^Comorbidity was measured using the Charlson Comorbidity Index (modified to exclude hemiplegia/paraplegia to avoid misclassifying MS complications) during the one-year period prior to the study entry date.^17,18^ The proportion of persons with MS scoring 1 or more on the Comorbidity Index is consistent with prior work^16^ conducted in similar cohorts. The most common comorbid conditions (present at study entry) which were identified using the Index were ‘chronic pulmonary disease’ (present in 1,102/15,350; 7.2% of the study cohort), ‘diabetes mellitus without chronic complications’ (1,011; 6.6%), and ‘cerebrovascular disease’ (629; 4.1%). Briefly, of the 25,698 ED visits with a known diagnosis, 37.4% (9,612/25,698) were made by MS cases with at least one comorbidity at study entry. For 96.2% (9,247/9,612) of these visits, an ED diagnosis code other than ‘MS’ was reported, of which 24.7% (2,282/9,247) led to hospitalization. For the 16,086 ED visits made by MS cases without comorbidity, an ED diagnosis code other than ‘MS’ was reported for 94.7% (15,226/16,086) of visits, of which 17.4% (2,648/15,226) led to hospitalization. Hence, regarding non-MS-related ED visits, there was a descriptive difference in the proportions of hospitalizations, but not in the proportions of all non-MS-related ED visits, between MS cases with and without comorbidity at study entry.

^d^Captured as prescriptions filled; some people were exposed to >1 DMD during follow, hence the sum of cases filling first and second generation DMD prescriptions exceeded the sum of cases ever filling a DMD prescription; first generation DMDs included beta-interferon and glatiramer acetate, second generation DMDs included natalizumab, fingolimod, dimethyl fumarate, teriflunomide, alemtuzumab, daclizumab and ocrelizumab. Pre-study entry (1-Jan-1996 to 31-Mar-2012) 19.5% (2,991/15,350) of MS cases had ever filled a DMD prescription.

^e^The denominator used to estimate the following proportions was the number of ED visits with a known diagnosis which led to hospitalization (n=5,238).
